# Principal component analysis of adipocytokines and insulin associate with risk factors of cardiovascular diseases

**DOI:** 10.1186/s13104-020-04976-9

**Published:** 2020-04-14

**Authors:** Habib Yarizadeh, Leila Setayesh, Moein Askarpoor, Sara Pooyan, Seyedeh Forough Sajjadi, Negin Badrooj, Caroline Roberts, Khadijeh Mirzaei

**Affiliations:** 1 Students’ Scientific Center, Tehran University of Medical Sciences, PO Box 1417755331, Tehran, Iran; 2 Department of Community Nutrition, School of Nutritional Sciences and Dietetics, Tehran University of Medical Sciences (TUMS), P.O. Box: 14155-6117, Tehran, Iran; 3 Department of Nutritional Sciences, School of Life Course Sciences, Faculty of Life Sciences and Medicine, King’s College London, London, UK

**Keywords:** Adipocytokines, Cardiovascular diseases, Obesity

## Abstract

**Objectives:**

Obesity plays an important role in the development of chronic diseases like cardiovascular diseases and diabetes. The possible underlying mechanism for this connection is that adipose tissue secretes an array of chemical messenger adipokines proinflammatory cytokines (tumor necrosis factor-alpha, interleukin-6, and interleukin-1-beta). This study aimed to investigate the linkage between adipocytokines and insulin with the cardiovascular disease risk, with particular reference to the adipokines galectin-3, plasminogen activator inhibitor-1, and interleukin-1-beta, C-reactive protein, and monocyte chemoattractant protein.

**Result:**

Two patterns were identified. The first pattern was galectin-3, plasminogen activator inhibitor-1 and interleukin-1-beta and the second one was C-reactive protein, insulin and monocyte chemoattractant protein-1. The second pattern was strongly associated with the higher scores for resting metabolic rate, diastolic blood pressure, homeostasis model insulin resistance index, lipid profile (except low density lipoprotein, total cholesterol), and body composition parameters (except fat free mass index and waist hip ratio), while negatively associated with age and high density lipoprotein level (all p < 0.05). The first pattern was, however, significantly associated with body fat mass, obesity degree percentage, waist circumference, fat mass index, and waist hip ratio (p < 0.05 for all).

This is a retrospective study. Ethics approval (IR.TUMS.VCR.REC.1395.1597).

## Introduction

Cardiovascular diseases (CVDs) is an umbrella term that includes diseases affecting the heart and blood vessels [1]. There are a number of modifiable (smoking, alcohol consumption, diet, exercise) and non-modifiable (genes) that may contribute to the onset and progression of CVD [2], with strong evidence suggesting obesity is a major risk factor for CVD. Relevant reports also demonstrated that obesity is predominant in CVD patients [3]. one possible underlying mechanism for this connection is that adipose tissue secretes an array of chemical messenger adipokines including leptin and proinflammatory cytokines [tumor necrosis factor-alpha (TNFα), interleukin-6 (IL-6), interleukin-1- beta (IL-1β)] [4]. Indeed, abnormal levels of adipokines may predispose obese individuals to cardiovascular diseases [5].

A high proportion of body fat mass, in particular abdominal fat is one of the major risk factors for CVD [6], and as a result, obesity is an independent underlying predictor of CVD which is often related to elevated levels of inflammatory mediators [7, 8]. These inflammatory mediators are regarded as independent risk factors for future coronary events [8]. Proinflammatory cytokines are capable of triggering the synthesis of C-reactive protein (CRP) by hepatocytes [9]. There is substantial evidence supporting the role of CRP in the pathogenesis and worsening of vascular inflammation, vessel damage and clinical CVD complications [10].

Obesity has become a burgeoning epidemic in the public health [11] and is inevitably accompanied by the increased level of plasma cholesterol, triglyceride, and blood sugar as well as levels of insulin, which can elevate susceptibility to the cardiovascular diseases [12]. The aim of this study was to determine if these factors contribute to cardiovascular disease in women.

This study aimed to investigate the linkage between serum markers of obesity and risk of cardiovascular diseases, with particular reference to the adipokines galectin-3, plasminogen activator inhibitor-1 (PAI-1), IL-1β, CRP, monocyte chemoattractant protein-1 (MCP-1), and insulin.

## Main text

### Method

#### Subjects

Three hundred and sixty women with body mass index (BMI) ≥ 25 were recruited from health centers of Tehran. Investigating obese individuals aged 18 to 50. Participants were excluded if they were taking medications or had history of type one diabetes mellitus. Smoking, alcohol consumption, pregnancy, lactation, and exercise were considered as additional exclusion criteria.

After an overnight (12 h) fast, volunteers attended to the clinical health centers and standard anthropometric parameters were recorded. Three readings were taken to calculate resting blood pressures at left brachial artery by an automated sphygmomanometer (Dinamap).

#### Procedures

Indirect calorimetry (spirometer METALYZERR 3B-R3, Cortex Biophysik GmbH, Leipzig, Germany) was used to calculate resting metabolic rate (RMR). To do so, the amount of consumed O_2_ and produced CO_2_ was measured after/when subjects had rested for at least 20 min. Gas ventilation was calibrated prior to each test.

#### Laboratory methods

A blood sample was also taken to assess serum concentrations of glucose (FBS), lipids, and adipokines (CRP, IL-1β) using enzyme-linked immunosorbent assay kits (R&D Systems, Techne Corporation, Minneapolis, MN) according to the manufacturer’s instructions. All samples were analyzed in duplicates. The homeostasis model insulin resistance index (HOMA) was computed as the product of fasting glucose and insulin level divided by 22.5 with molar unit (mmol/L).

#### Statistical analysis

All statistical analysis was conducted by the SPSS version 22 (SPSS Inc., Chicago, IL). Data are presented as arithmetic means plus their 95% confidence interval (CI) and standard deviations unless otherwise stated.

Factor (principal component) analysis was performed to determine which plasma concentration of adipokines associated with most of observed variations. Two patterns were identified as shown in Additional file 1: Table S1. The significance of differences between low and high value of each pattern and CVD risk factors were computed by analysis of variance (ANOVA) (Additional file 2: Table S2). In addition, to keep uncorrelated factors and greater interpretability, the patterns were rotated and normalized using Varimax transformation method and Kaiser Method, respectively. Pearson’s correlation coefficient indicates the association between each adipokine pattern score and CVD risk factors (Additional file 3: Table S3). Multiple linear regression analysis was followed to calculate the incidence and strength of associations (Tables 1 and 2). Two-sided p values < 0.05 were considered as statistically significant. Confounders including age, physical activity, energy intake, and BMI were used to adjust for linear regression analysis.

**Table 1:** Association between demographics, medical and biomedical characteristics with patterns 1 of Adipokines

Variable	Pattern 1 group			
	B	95.0% confidence interval	${\text{p}}\_{\text{value}}_{\upalpha}$	${\text{p}}\_{\text{value}}_{\beta }$
Age (years)	− 0.12	− 0.81 to 0.56	0.71	0.71
Weight (kg)	− 0.92	− 1.87 to 0.02	0.05	0.05
BMI (kg/m^2^)	− 0.34	− 0.63 to − 0.04	0.02	0.06
RMR and blood pressure parameters				
RMR (kcal/day)	− 15.58	− 36.94 to 5.78	0.15	0.13
SBP (mmHg)	− 0.89	− 2.08 to 0.28	0.13	0.11
DBP (mmHg)	− 0.93	− 1.72 to − 0.14	0.02	0.06
Blood parameters				
Cholesterol (g/dl)	− 0.26	− 5.42 to 0.89	0.15	0.13
TG (g/dl)	0.11	− 5.20 to 5.43	0.96	0.94
LDL (mg/dl)	− 1.30	− 1.35 to 0.90	0.24	0.17
HDL (mg/dl)	− 0.60	− 1.49 to 0.28	0.18	0.14
hs.CRP (mg/dl)	0.10	− 0.28 to 0.46	0.57	0.60
HOMA	0.01	− 0.11 to 0.14	0.83	0.81
FBS (mmol)	0.03	− 0.02 to 0.08	0.23	0.15
Body composition parameters				
BFM (kg)	− 0.98	− 1.60 to − 0.37	0.002	0.001
FFM (kg)	− 0.03	− 0.48 to 0.41	0.86	0.85
SLM (kg)	− 0.00	− 0.43 to 0.42	0.98	0.97
SMM (kg)	− 0.01	− 0.27 to 0.25	0.91	0.93
ODP	− 1.57	− 2.98 to − 0.15	0.03	0.02
WC (cm)	− 1.07	− 1.82 to − 0.32	0.005	0.005
FFMI	− 0.13	− 0.77 to − 0.50	0.68	0.67
FMI	− 0.37	− 0.61 to − 0.13	0.003	0.002
WHR	− 0.00	− 0.01 to − 0.00	0.004	0.004

Mean ± SD: mean ± standard deviation; p-value_α: p-value result from ANOVA; p-value_β p: p-value result from Generalized Linear mode (General linear model for adjusting age, gender, and total energy intake) l; BFM: body fat mass; SLM: soft lean mass; ODP: obesity degree percentage; BMI: body mass index; TG: triglyceride; LDL: low density lipoprotein; HDL: high density lipoprotein; FFM: fat free mass; RMR: resting metabolic rate; SBP: systolic blood pressure; DBP: diastolic blood pressure; HOMA: homeostasis model insulin resistance index; hs-CRP: high sensitivity C-reactive protein; WC: waist circumference; WHR: waist hip ratio; FFMI: fat free mass index; FMI: fat mass index

**Table 2:** Association between demographics, medical and biomedical characteristics with patterns 2 of adipokines

Variable	Pattern 2 group			
	B	95.0% confidence interval	${\text{p}}\_{\text{value}}_{\upalpha}$	${\text{p}}\_{\text{value}}_{\beta }$
Age (years)	− 0.88	− 1.57 to − 0.20	0.010	0.012
Weight (kg)	1.57	0.59 to 2.47	0.001	0.003
BMI (kg/m^2^)	0.48	0.18 to 0.77	0.002	0002
RMR and blood pressure parameters				
RMR (kcal/day)	42.12	21.62 to 62.63	0.000	0.000
SBP (mmHg)	0.67	− 0.49 to 1.87	0.25	0.21
DBP (mmHg)	1.88	0.60 to 2.14	0.00	0.001
Blood parameters				
Cholesterol (g/dl)	0.93	− 2.24 to 4.12	0.56	0.52
TG (g/dl)	8.44	3.18 to 13.70	0.002	0.002
LDL (mg/dl)	1.28	− 0.93 to 3.50	0.25	0.17
HDL (mg/dl)	− 0.164	− 2.53 to − 0.76	0.000	0.000
hs.CRP (mg/dl)	2.11	1.82 to 2.41	0.000	0.000
HOMA	0.58	0.48 to 0.69	0.000	0.000
FBS (mmol)	0.02	− 0.02 to 0.07	0.30	0.36
Body composition analysis				
BFM (kg)	0.78	0.16 to 1.40	0.013	0.012
FFM (kg)	0.61	0.16 to 1.05	0.007	0.009
SLM (kg)	0.62	0.20 to 1.04	0.004	0.005
SMM (kg)	0.38	0.12 to 0.65	0.004	0.005
ODP	2.23	0.82 to 3.64	0.002	0.002
WC (cm)	1.15	0.40 to 1.90	0.003	0.003
FFMI	− 0.08	− 0.73 to 0.55	0.78	0.77
FMI	0.26	0.02 to 0.51	0.03	0.026
WHR	0.004	0.000 to 0.008	0.084	0.078

Mean ± SD: mean ± standard deviation; p-value_α: p-value result from ANOVA; p-value_β p: p-value result from Generalized Linear mode (General linear model for adjusting age, gender, and total energy intake) l; BFM: body fat mass; SLM: soft lean mass; ODP: obesity degree percentage; BMI: body mass index; TG: triglyceride; LDL: low density lipoprotein; HDL: high density lipoprotein; FFM: fat free mass; RMR: resting metabolic rate; SBP: systolic blood pressure; DBP: diastolic blood pressure; HOMA: homeostasis model insulin resistance index; hs-CRP: high sensitivity C-reactive protein; WC: waist circumference; WHR: waist hip ratio; FFMI: fat free mass index; FMI: fat mass index

### Results

#### Subject characteristics

Table 3 depicts main clinical and biochemical characteristics of the subjects. Mean age ranged from 18 to 50 years and BMI ranged between 25 and 40.70 kg/m^2^.

**Table 3:** Characteristics of the study participants

Variable	Mean	SD	Minimum	Maximum
Age (years)	36.52	8.32	18.00	50.00
Body weight (kg)	78.75	11.51	1.00	119.50
BMI (kg/m^2^)	30.33	3.65	25.00	40.70
RMR and blood pressure parameters				
RMR (kcal/day)	1566.51	253.49	952.0	2467.00
SBP (mmHg)	113.11	14.05	76.00	173.00
DBP (mmHg)	78.13	9.36	51.00	111.00
Blood parameters				
Serum cholesterol (g/dl)	185.61	38.48	104.00	433.00
TG (g/dl)	118.39	64.54	37.00	512.00
LDL (mg/dl)	96.98	26.86	34.00	282.00
HDL (mg/dl)	47.77	10.87	18.00	84.00
Hs.CRP (mg/dl)	4.05	4.54	0.00	22.73
FBS (mmol)	4.92	0.63	3.72	11.22
HOMA	3.33	1.52	0.39	16.59
Body composition analysis				
BFM (Kg)	33.43	7.60	19.40	53.20
FFM (Kg)	46.62	5.43	35.30	67.70
SLM (Kg)	43.84	5.16	26.10	63.80
SMM (Kg)	25.59	3.22	18.90	37.90
ODP	142.86	17.33	116.00	189.00
WC (cm)	98.45	9.24	80.10	123.20
FFMI	18.32	7.78	14.60	147.80
FMI	12.91	2.96	6.90	21.20
WHR	0.93	0.05	0.81	1.08

*SD* standard deviation, *BFM* body fat mass, *SLM* soft lean mass, *ODP* obesity degree percentage, *BMI* body mass index, *TG* triglyceride, *LDL* low density lipoprotein, *HDL* high density lipoprotein, *FFM* fat free mass, *RMR* resting metabolic rate, *SBP* systolic blood pressure, *DBP* diastolic blood pressure, *HOMA* homeostasis model insulin resistance index, *hs-CRP* high sensitivity C-reactive protein, *WC* waist circumference, *WHR* waist hip ratio, *FFMI* fat free mass index, *FMI* fat mass index

#### Correlation analysis

Single correlation of each adipokines with clinical and biochemical risk factors of CVD are detailed in Additional file 3: Table S3. The adipokines galectin-3 appeared related to BMI (r = − 0.109, p = 0.038), diastolic blood pressure (DBP) (r = − 0.123, p = 0.023), body fat mass (BFM) (r = − 0.174, p = 0.003), ODP (r = − 0.119, p = 0.044), waist circumference (WC) (r = − 0.140, p = 0.017), FMI (r = − 0.177, p = 0.003), as well as waist-hip ratio (WHR) (r = − 0.140, p = 0.017). Similar results were obtained between PAI-1 and BMI (r = − 0.111, p = 0.036), DBP (r = − 0.141, p = 0.009), BFM (r = − 0.177, p = 0.003), ODP (r = − 0.120, p = 0.041), WHR (r = − 0.155, p = 0.008), FMI (r = − 0.176, p = 0.003), plus WHR (r = − 0.166, p = 0.005). In case of CRP, the significant correlations were observed with BMI (r = 0.121, p = 0.022), DBP (r = 0.129, p = 0.018), HDL (r = − 0.152, p = 0.007), hs.CRP (r = 0.858, p = 0.0001), HOMA (r = 0.167, p = 0.004), and ODP (r = − 0.133, p = 0.024). Neither IL-1β nor MCP-1 were significantly associated with any of the anthropocentric data, blood and body composition parameters except for a positive association between IL-1β and age (r = 0.128, p = 0.015) and negative correlation between MCP-1 and FBS (r = − 0.147, p = 0.009).

On the other hand, insulin shown very broad correlations with age (r = − 0.146, p = 0.006), weight (r = 0.240, p = 0.0001), BMI (r = 0.232, p = 0.0001), RMR (r = 0.317, p = 0.0001), SBP (r = 0.112, p = 0.039), DBP (r = 0.217, p = 0.0001), TG (r = 0.290, p = 0.0001), HDL (r = − 0.145, p = 0.01), hs.CRP (r = 0.178, p = 0.001), HOMA (r = 0.880, p = 0.0001), FBS (r = 0.176, p = 0.002), BFM (r = 0.227, p = 0.0001), fat-free mass (FFM) (r = 0.292, p = 0.0001), soft lean mass (SLM) (r = 0.278, p = 0.0001), SMM (r = 0.307, p = 0.0001), ODP (r = 0.253, p = 0.0001), WC (r = 0.307, p = 0.0001), FMI (r = 0.175, p = 0.003), WHR (r = 0.240, p = 0.0001).

Two patterns were identified. The first pattern was characterized by high level of galectin-3 and, PAI-1 and low level of IL-1β. The second one was associated with CRP, insulin and MCP-1 (Additional file 1: Table S1). As shown in Table 2, association analysis of adipokines patterns demonstrated that pattern 2 were strongly correlated with the higher scores for RMR, DBP, HOMA, lipid profile, and body composition parameters, while negatively associated with age and HDL level (all p < 0.05). However, SBP, cholesterol, LDL, FBS, FFMI, and WHR appeared unrelated to PCA2 (all p > 0.05).

In contrast, neither biochemical parameters nor blood pressure of participants was significantly correlated to PCA1 pattern (all p > 0.05). The first pattern was, however, significantly associated with BFM, ODP, WC, FMI, and WHR (p < 0.05 for all).

### Discussion

This study aimed to investigate the association between adipocytokines and insulin patterns with the risk of cardiovascular diseases. In this study we identified two separate adipokines patterns, explaining 56% of the total variance in the data. Of these, pattern 1 characterized by the high level of galectin-3, and PAI-1 and low concentration of IL-1β. The second one included CRP, MCP-1, plus insulin. In the linear regression model derived from our data, the link between the second pattern and body composition parameters, and biochemical markers of CVD was yielded significant p-values except for SBP, LDL, and FBS.

Previous investigations on the link of adipokines and CVD risk factors have been mostly limited to single adipokines [13, 14]. But in physiology, a complex network of chemical messengers contributes to the CVD [15]. Thus, identification and evaluation of these factors in combination with each other could, at least partly, open a clear window toward prediction of the CVD. To do so, PCA was employed as a powerful statistical tool to recognize potential contributors to the observed risk factors [16]. Next, univariate regression performed to provide detailed information about quantitative associations between adipokines level and biological outcomes of the interest.

Considering the number of independent variables (adipokines plus insulin) included in this study, PCA was preferentially used for data reduction by taking a linear combination of the existing 6 orthogonal variables, allowing us to identify potentially significant patterns of adipokines plus insulin associated with CVD risk factors [17].

In consensus with the literature, our results showed that CRP, MCP-1, and insulin may be the key determinants of CVD predisposition, suggesting inflammation plays a key role in CVD development. Unraveling the molecular mechanism behind these associations may elucidate their potential for prediction of the cardiovascular events. In this respect, obesity has already been associated with low-grade inflammation in humans [18, 19]. The exact trigger is unknown yet [20], however, a number of systemic chemical messengers particularly resistin are released into the bloodstream [21]. Resistin (also called adipose tissue-specific secretory factor) is a hormone derived from adipocytes and macrophages that could significantly contribute to inflammation and CVD [22]. Resistin has been demonstrated to increase LDL levels and contribute to insulin resistance [23, 24]. It also upregulates expression of an inflammatory component called MCP-1 [25]. MCP-1 is produced by multiple cell types, including fibroblasts, monocytes, mesangial and also endothelial cells either constitutively or after induction by oxidative stress [26]. Resistin recruits monocytes and T cells to the sites of inflammation or damaged tissues [27]. Migrated monocytes differentiate into the foam cells in sub endothelial space where they initiate to produce fatty streak formations that result in atherosclerotic plaque formation [28]. Some other monocytes differentiate into tissue-resident macrophages with the ability to produce proinflammatory cytokines TNF-α, IL-1, and IL-6 that affect the liver, heart, bones, kidney, and many other tissues [29, 30]. Of these, the liver releases acute phase proteins like CRP in response to mild inflammation. CRP is believed to cause inflammation on coronary vessels, subsequently leading to myocardial ischemia [31]. On the other hand, long-term elevation of insulin level during obesity, prolongs obesity, creating a positive feedback loop and inflammation changes into the chronic form. Insufficient reperfusion further exacerbates the inflammation in injured myocardium and causes myocardial necrosis [32].

### Conclusion

Our findings support the hypothesis of multiple determinants in the prediction of CVD risk factors in obese adult females. The utility of PCA helped to statistically analyze a comprehensive panel of adipokines and identifying potential candidates for more detailed studies in the future. Based on the results of the PCA, the panel of CRP, MCP-1, and insulin were shown responsible for the most significant variability in cardiovascular disease risk factors, however, further investigations are required to confirm whether our adipokines patterns are associated with the clinical and biological.

## Limitations

To the best of our knowledge, this was the first study to investigate the adipokines pattern and CVD, however there were a number of limitations. The study was limited to the number of participants and same-sex samples. We only recruited obese females, therefore our results may not be applicable to the general population. Furthermore, we had a small number of predominantly inflammatory adipokines and further studies should look at introducing more adipokines another important limitation is the cross-sectional design of the study that prevented us from inferring causality.

## Supplementary information

**Additional file 1: Table S1.** Principal factor loading of adipokines.

**Additional file 2: Table S2.** Demographic, medical and biomedical characteristics with adherence to two groups of Adipokines patterns.

**Additional file 3: Table S3.** Correlation between demographics, medical and biomedical characteristics with all of Adipokines and insulin.

## Data Availability

Since the privacy of research participants may be compromised, we cannot make the information publicly available.

## Abbreviations

CVDs: Cardiovascular diseases
TNFα: Tumor necrosis factor-alpha
IL-6: Interleukin-6
IL-1β: Interleukin-1- beta
CRP: C-reactive protein
PAI-1: Plasminogen activator inhibitor-1
MCP-1: Monocyte chemoattractant protein-1
BMI: Body mass index
RMR: Resting metabolic rate
FBS: Fasting blood sugar
CI: Confidence interval
ANOVA: Analysis of variance
SBP: Systolic blood pressure
DBP: Diastolic blood pressure
BFM: Body fat mass
ODP: Obesity degree percentage
WC: Waist circumference
WHR: Waist-hip ratio
FFM: Fat-free mass
SLM: Soft lean mass
