# Supplementary material for: Principal component analysis of adipocytokines and insulin associate with risk factors of cardiovascular diseases
Source: BMC Res Notes. 2020 Apr 14;13:212. doi: 10.1186/s13104-020-04976-9 (PMC7157993; doi:10.1186/s13104-020-04976-9)
Supplement: Supplementary file 1 — Additional file 1: Table S1. Principal factor loading of adipokines. [file 13104_2020_4976_MOESM1_ESM.docx]

| **Table S1: Principal factor loading of adipokines** | | | | |
| --- | --- | --- | --- | --- |
|  | Component | |  |  |
|  | P1 | P2 |  |  |
| Galectin-3 | 0.959 |  |  |  |
| PAI-1 | 0.958 |  |  |  |
| IL-1β | -0.525 |  |  |  |
| *CRP* |  | 0.714 |  |  |
| Insulin |  | 0.624 |  |  |
| MCP-1 |  | 0.554 |  |  |
| Percent of variance explained | 35.58 | 20.72 |  |  |
| *All Adipokines are adjusted for visceral fat by residual method. Rotation Method: Varimax with Kaiser Normalization. Rotation converged in 3 iterations*  *PAI-1: plasminogen activator inhibitor-1; IL-1β: interleukin-1 beta; CRP: C-reactive protein; MCP-1: Monocyte chemoattractant protein-1;* | | | |  |
