# Supplementary material for: Principal component analysis of adipocytokines and insulin associate with risk factors of cardiovascular diseases
Source: BMC Res Notes. 2020 Apr 14;13:212. doi: 10.1186/s13104-020-04976-9 (PMC7157993; doi:10.1186/s13104-020-04976-9)
Supplement: Supplementary file 2 — Additional file 2: Table S2. Demographic, medical and biomedical characteristics with adherence to two groups of Adipokines patterns. [file 13104_2020_4976_MOESM2_ESM.docx]

**Table S2: Demographic, medical and biomedical characteristics with adherence to two groups of Adipokines patterns**

| Variable | Pattern 1 group | | | Pattern 2 group | | |
| --- | --- | --- | --- | --- | --- | --- |
|  | Low  Mean±SD | High  Mean±SD | ${p\_value}_{\alpha}$ | Low  Mean±SD | High  Mean±SD | ${p\_value}_{\alpha}$ |
| Age (years) | 36.27±8.61 | 36.68±8.13 | 0.65 | 37.37±7.83 | 35.92±8.61 | 0.10 |
| Weight (kg) | 78.95±11.84 | 78.62±11.31 | 0.79 | 77.97±10.14 | 79.30±12.37 | 0.28 |
| BMI (kg/m^2^) | 30.43±3.49 | 30.26±3.75 | 0.65 | 30.04±3.44 | 30.54±3.78 | 0.20 |
| RMR and blood pressure parameters | | | | | | |
| RMR (kcal/d) | 1557.83±233.04 | 1575.00±272.59 | 0.57 | 1510.20±228.31 | 1626.51±265.78 | 0.000 |
| SBP (mmHg) | 112.24±13.28 | 113.71±14.56 | 0.34 | 110.92±14.27 | 114.67±13.71 | 0.01 |
| DBP (mmHg) | 78.76±10.17 | 77.69±8.75 | 0.29 | 76.43±9.84 | 79.34±8.83 | 0.00 |
| Blood parameters | | | | | | |
| Cholesterol (g/dl) | 186.47±36.33 | 184.90±40.21 | 0.71 | 181.1655±33.92 | 189.06±41.44 | 0.06 |
| TG (g/dl) | 118.30±65.12 | 118.46±64.25 | 0.98 | 109.22±61.29 | 125.51±66.25 | 0.02 |
| LDL (mg/dl) | 95.75±24.49 | 97.98±28.66 | 0.46 | 92.93±23.86 | 100.13±28.64 | 0.01 |
| HDL (mg/dl) | 47.12±11.17 | 48.29±10.61 | 0.34 | 48.50±10.93 | 47.20±10.81 | 0.29 |
| hs.CRP (mg/dl) | 3.79±3.97 | 4.26±4.95 | 0.35 | 2.00±1.85 | 5.66±5.31 | 0.000 |
| HOMA | 3.39±1.13 | 3.27±1.81 | 0.49 | 2.81±0.88 | 3.78±1.81 | 0.000 |
| FBS (mmol) | 4.84±0.53 | 4.98±0.69 | 0.05 | 4.81±0.51 | 5.00±0.70 | 0.01 |
| Body composition analysis | | | | | | |
| BFM (kg) | 33.28±7.01 | 33.57±8.16 | 0.74 | 32.29±7.40 | 34.63±7.64 | 0.00 |
| FFM (kg) | 46.13±5.08 | 47.11±5.73 | 0.12 | 45.95±4.92 | 47.34±5.86 | 0.03 |
| SLM (kg) | 43.26±4.87 | 44.41±5.40 | 0.05 | 43.09±4.71 | 44.64±5.52 | 0.01 |
| SMM (kg) | 25.27±3.038 | 25.91±3.37 | 0.39 | 25.14±2.90 | 26.07±3.47 | 0.01 |
| ODP | 141.48±16.28 | 144.23±18.27 | 0.17 | 139.69±16.02 | 146.23±18.08 | 0.00 |
| WC (cm) | 98.11±8.57 | 98.78±9.89 | 0.53 | 96.70±8.94 | 100.30±9.23 | 0.00 |
| FFMI | 18.55±10.97 | 18.08±1.39 | 0.60 | 18.49±10.74 | 18.12±1.48 | 0.68 |
| FMI | 12.86±2.87 | 12.95±3.06 | 0.78 | 12.47±2.85 | 13.37±3.02 | 0.00 |
| WHR | 0.93±0.04 | 0.92±0.05 | 0.62 | 0.92±0.05 | 0.93±0.04 | 0.01 |

*Mean± SD: mean ± standard deviation; P-value_α: p-value result from ANOVA; BFM: Body fat mass; SLM: Soft lean mass; ODP: Obesity degree percentage; BMI: Body mass index; TG: Triglyceride; LDL: Low density lipoprotein; HDL; High density lipoprotein; FFM: fat free mass;* *RMR, resting metabolic rate; SBP: systolic blood pressure; DBP: diastolic blood pressure; HOMA: homeostasis model insulin resistance index; hs-CRP:* *high sensitivity C-reactive protein; WC: Waist Circumference; WHR: Waist hip ratio; FFMI: fat free mass index; FMI: fat mass index.*
