# Supplementary material for: Principal component analysis of adipocytokines and insulin associate with risk factors of cardiovascular diseases
Source: BMC Res Notes. 2020 Apr 14;13:212. doi: 10.1186/s13104-020-04976-9 (PMC7157993; doi:10.1186/s13104-020-04976-9)
Supplement: Supplementary file 3 — Additional file 3: Table S3. Correlation between demographics, medical and biomedical characteristics with all of Adipokines and insulin. [file 13104_2020_4976_MOESM3_ESM.docx]

**Table S3**: correlation between demographics, medical and biomedical characteristics with all of Adipokines and insulin.

| Variable | IL1β | | MCP-1 | | Galectin-3 | | CRP | | insulin | | PAI-1 | |
| --- | --- | --- | --- | --- | --- | --- | --- | --- | --- | --- | --- | --- |
|  | r | P value | r | P value | r | P value | r | P value | r | P value | r | P value |
| Age (years) | -0.018 | 0.728 | -0.080 | 0.132 | -0.033 | 0.532 | -0.033 | 0.536 | -0.146 | 0.006 | -0.035 | 0.512 |
| Weight (Kg) | 0.128 | 0.015 | -0.009 | 0.865 | -0.069 | 0.194 | 0.066 | 0.209 | 0.240 | 0.000 | -0.077 | 0.147 |
| BMI (Kg/m^2^) | 0.079 | 0.136 | -0.046 | 0.382 | -0.109 | 0.038 | 0.121 | 0.022 | 0.232 | 0.000 | -0.111 | 0.036 |
| RMR and blood pressure parameters | | | | | | | | | | | | |
| RMR (kcal/d) | 0.089 | 0.133 | 0.011 | 0.851 | -0.071 | 0.231 | 0.110 | 0.065 | 0.317 | 0.000 | -0.058 | 0.330 |
| SBP (mmHg) | -0.011 | 0.846 | 0.008 | 0.890 | -0.087 | 0.107 | 0.022 | 0.680 | 0.112 | 0.039 | -0.093 | 0.085 |
| DBP (mmHg) | 0.019 | 0.725 | 0.038 | 0.480 | -0.123 | 0.023 | 0.129 | 0.018 | 0.217 | 0.000 | -0.141 | 0.009 |
| Blood parameters | | | | | | | | | | | | |
| Cholesterol (g/dl) | 0.026 | 0.649 | -0.066 | 0.244 | -0.085 | 0.132 | 0.027 | 0.637 | 0.101 | 0.072 | -0.081 | 0.148 |
| TG (g/dl) | -0.021 | 0.711 | -0.058 | 0.302 | 0.005 | 0.925 | 0.102 | 0.068 | 0.290 | 0.000 | -0.009 | 0.870 |
| LDL (mg/dl) | 0.021 | 0.709 | -0.019 | 0.731 | -0.061 | 0.281 | 0.082 | 0.142 | 0.057 | 0.313 | -0.079 | 0.160 |
| HDL (mg/dl) | -0.016 | 0.779 | -0.073 | 0.195 | -0.106 | 0.059 | -0.152 | 0.007 | -0.145 | 0.010 | -0.074 | 0.187 |
| hs.CRP (mg/dl) | -0.014 | 0.806 | 0.094 | 0.096 | 0.022 | 0.701 | 0.858 | 0.000 | 0.178 | 0.001 | -0.007 | 0.908 |
| HOMA | 0.001 | 0.984 | 0.002 | 0.974 | 0.047 | 0.426 | 0.167 | 0.004 | 0.880 | 0.000 | 0.019 | 0.742 |
| FBS (mmol) | -0.073 | 0.196 | -0.147 | 0.009 | 0.053 | 0.350 | 0.072 | 0.202 | 0.176 | 0.002 | 0.045 | 0.428 |
| Body composition parameters | | | | | | | | | | | | |
| BFM (kg) | 0.094 | 0.109 | -0.026 | 0.661 | -0.174 | 0.003 | 0.079 | 0.179 | 0.227 | 0.000 | -0.177 | 0.003 |
| FFM (kg) | 0.065 | 0.274 | 0.009 | 0.879 | 0.019 | 0.746 | -0.005 | 0.939 | 0.292 | 0.000 | 0.013 | 0.822 |
| SLM (kg) | 0.068 | 0.246 | 0.028 | 0.640 | 0.028 | 0.635 | 0.012 | 0.840 | 0.278 | 0.000 | 0.023 | 0.693 |
| SMM (kg) | 0.064 | 0.279 | 0.000 | 1.000 | 0.025 | 0.671 | 0.008 | 0.894 | 0.307 | 0.000 | 0.017 | 0.775 |
| ODP | 0.086 | 0.145 | -0.052 | 0.379 | -0.119 | 0.044 | 0.133 | 0.024 | 0.253 | 0.000 | -0.120 | 0.041 |
| WC (cm) | 0.115 | 0.050 | -0.051 | 0.387 | -0.140 | 0.017 | 0.072 | 0.220 | 0.307 | 0.000 | -0.155 | 0.008 |
| FFMI | 0.018 | 0.758 | -0.016 | 0.784 | -0.020 | 0.740 | -0.048 | 0.421 | 0.035 | 0.555 | -0.020 | 0.737 |
| FMI | 0.083 | 0.161 | -0.043 | 0.469 | -0.177 | 0.003 | 0.110 | 0.063 | 0.175 | 0.003 | -0.176 | 0.003 |
| WHR | 0.111 | 0.060 | -0.057 | 0.337 | -0.140 | 0.017 | 0.008 | 0.894 | 0.240 | 0.000 | -0.166 | 0.005 |

*R: correlation; BFM: Body fat mass; SLM: Soft lean mass; ODP: Obesity degree percentage; BMI: Body mass index; TG: Triglyceride; LDL: Low density lipoprotein; HDL; High density lipoprotein; FFM: fat free mass;* *RMR, resting metabolic rate; SBP: systolic blood pressure; DBP: diastolic blood pressure; HOMA: homeostasis model insulin resistance index; hs-CRP:* *high sensitivity C-reactive protein; WC: Waist Circumference; WHR: Waist hip ratio; FFMI: fat free mass index; FMI: fat mass index.*
